# Prevalence and possible factors of cognitive frailty in the elderly with hypertension and diabetes

**DOI:** 10.3389/fcvm.2022.1054208

**Published:** 2022-11-21

**Authors:** Shourong Lu, Qiao Xu, Jie Yu, Ying Yang, Zhuo Wang, Bingshan Zhang, Shuqiang Wang, Xiaorong Chen, Yunyun Zhang, Xiaowei Zhu, Kan Hong

**Affiliations:** ^1^Department of Geriatric, The Affiliated Wuxi People's Hospital of Nanjing Medical University, Wuxi, China; ^2^Department of Medicine, Wuxi Xin'an Community Health Service Center, Wuxi, China; ^3^Department of General Practice, The Affiliated Wuxi People's Hospital of Nanjing Medical University, Wuxi, China; ^4^Department of Endocrinology, The Affiliated Wuxi People's Hospital of Nanjing Medical University, Wuxi, China

**Keywords:** diabetes, hypertension, cognitive frailty, elderly, activities of daily living

## Abstract

**Background:**

Cognitive frailty is the coexistence of physical frailty and mild cognitive impairment. Research shows that cognitive frailty is related to an increased risk of hospitalization, mortality, disability, and dementia. Diabetes and hypertension are common risk factors for physical frailty and cognitive impairment. However, the factors influencing cognitive frailty in the elderly with hypertension and diabetes are still unclear. This study aimed to investigate the possible factors influencing cognitive frailty in the elderly with hypertension and diabetes.

**Methods:**

A cross-sectional study was conducted. We evaluated people over 60 years with hypertension and diabetes who underwent physical examination in Wuxi Xin'an Community Health Service Center. Frail scale, Montreal Cognitive Assessment-Basic and clinical dementia rating were used to assess cognitive frailty. We collected demographic characteristics, hypertension and diabetes-related laboratory indicators of the participants. We also used various scales to assess the overall health status of the elderly.

**Results:**

Approximately 20.8% of the participants were determined to have cognitive frailty in elderly adults with hypertension and diabetes. These participants were older, had a lower monthly income, and included a higher proportion of peasants. They also had a higher level of depression (*p* = 0.037), higher risk of falls (*p* = 0.000), higher risk of malnutrition (*p* = 0.002), poorer ability to perform activities of daily living (ADL) (*p* = 0.000), and less social support (*p* = 0.030). Multivariate regression analysis was used to further assess the factors for cognitive frailty. After adjusting for possible confounders, age and ADL score emerged as risk factors, whereas high monthly income decreased the risk of cognitive frailty.

**Conclusion:**

Cognitive frailty is correlated with age, income, and ability to perform daily living activities in the elderly with diabetes and hypertension. Closer attention to the elderly who have low income and poor self-care ability may play an important role in the early prevention of cognitive frailty and even dementia.

## Introduction

Cognitive frailty, defined as the co-existence of physical frailty and cognitive impairment, has recently attracted increasing attention. The term “cognitive frailty” was coined by experts in 2013 (1). However, its definition has never been well-established. Researchers in different regions of the world have different definitions. Assessment scales are also different. The Fried scale and Frail scale are commonly used to evaluate physical frailty. Montreal Cognitive Assessment (MoCA) scale and Mini-Mental State Examination (MMSE) scale are commonly used in cognitive assessment. Ruan et al. (2) classify cognitive frailty into two types: reversible and potentially reversible. This study considered the latter definition. It is defined by the presence of physical frailty or prefrailty and cognitive impairment, excluding dementia, caused by various reasons (2). The prevalence of cognitive frailty in community settings is 1.2 to 7.7%, which increases to 20% in nursing centers and inpatient departments (3–6).

Cognitive frailty is closely associated with a higher risk of hospitalization, death, and disability (7, 8). It is also a strong predictor of overall dementia and vascular dementia (9). Thus, early intervention for the population with cognitive frailty can help prevent dementia and other aggravations. As we all know, cognitive frailty is strongly correlated with multiple factors. Panza et al. mentioned some possible neurobiological mechanisms underlying cognitive frailty, including vascular diseases, sarcopenia, metabolic disorders, nutritional status, psychological factors and inflammatory status (10). Due to reduced ability of activities and decline of brain function, the prevalence of cognitive frailty increases with age (11). Weight loss and vitamin deficiency may lead to physical frailty. Several studies have shown that the incidence of malnutrition is extremely high among elderly people with cognitive frailty (12–14). In addition, level of education is strongly associated with cognitive decline. The longer the years of education, the slower the cognitive decline (15, 16). Older adults with depression are more likely to develop cognitive frailty (17). Liu et al. indicates that moderate physical activities for 1 year can reduce the progression of cognitive frailty in sedentary older adults (18).

Hypertension and diabetes, the most common chronic diseases worldwide (19, 20), have been considered risk factors for physical frailty (21, 22) and cognitive impairment (23, 24). Thus, more attention should be paid to older adults with hypertension and diabetes. This study aimed to investigate the risk factors of cognitive frailty in the elderly with hypertension and diabetes.

## Materials and methods

### Participants and data collection

A cross-sectional study was conducted in Wuxi Xin'an Community Health Service Center from April 2018 to May 2018. Participants included older adults with diabetes and hypertension.

Inclusion criteria: 1. aged over 60; 2. previous diagnosis of hypertension and diabetes.

Exclusion criteria: 1. dementia; 2. severe hepatic and renal insufficiency; 3. failure to cooperate in the assessment using various scales.

The demographic and clinical variables included age, gender, occupation, education level, income per month, smoking history, and drinking history. Serum albumin, calcium, 25- hydroxy Vitamin D, triglycerides (TG), low-density lipoprotein cholesterol (LDL), high-density lipoprotein cholesterol (HDL), homocysteine, folic acid, Vitamin B12, fasting plasma glucose, fasting insulin, and HbA1c levels were assessed by the Laboratory Center of Wuxi People's Hospital.

### Assessment of cognitive function

Montreal Cognitive Assessment-Basic (MoCA-B) was used to evaluate cognitive function. MoCA-B score <26 and clinical dementia rating (CDR) = 0.5 (excluding dementia) indicated mild cognitive impairment.

### Other evaluation scales

The 5-item Frail scale was used to assess physical frailty. Frail score ≥1 indicated physical frailty, with a higher score showing a higher level of frailty. The Mini-Nutrition Assessment-Short Form (MNA-SF) evaluated the nutritional status of the participants. MNA-SF scores <8, 8–11, and >11 indicated malnutrition, risk of nutrition, and no malnutrition, respectively. The Geriatric Depression Scale 15-item (GDS-15) assessed the psychological health of participants, with a higher score indicating severe depression. The 10-item Social Support Rating Scale (SSRS) evaluated support from themselves, others and society. A higher total score indicated better social support. The Morse Fall Scale (MFS) was used to assess participants' risk of falling, with MFS scores <25, 25–45, and >45 indicating low risk, medium risk, and high risk, respectively. The ability to perform activities of daily living (ADL) was assessed at two levels. The basic activities of daily living scale (BADL) assessed skills including eating, dressing, grooming, bathing, going to the toilet, and walking; the instrumental activities of daily living scale (IADL) evaluated skills such as making phone calls, shopping, preparing meals, doing housework, washing clothes, taking public transport, taking drugs, and money management. A score >14 showed functional decline, with a higher score indicating decreased ability to perform daily living activities.

### Statistical analysis

All variables were tested for normal distribution. Student *t*-test, Mann-Whitney U test, and Chi-square test were conducted to compare the variables between groups. Continuous variables with normal distribution were presented as mean with standard deviation. Continuous variables with non-normal distribution were presented as median with quartile, and categorical variables as percentages. The multivariate logistic regression analysis was also performed. SPSS 22.0 software was used for data analysis, and *p* < 0.05 was considered statistically significant.

## Results

A total of 154 participants were enrolled, including 91 females (59.1%), 98 with a primary level or below education (63.6%), and 122 non-drinkers (79.2%). After the assessment, 32 (20.8%) were determined to have cognitive frailty. These participants were older, had a lower monthly income, and included a higher proportion of peasants. Participants with cognitive frailty also had higher scores of GDS-15 (*p* = 0.037), higher risk of malnutrition (*p* = 0.002), higher fall risk (*p* = 0.000), higher ADL score (*p* = 0.000), poorer daily living ability (*p* = 0.000), and less social support (*p* = 0.030) (Table 1). The participants between the two groups did not differ in laboratory indexes, such as lipid levels, albumin, calcium, 25- hydroxy Vitamin D, homocysteine, folic acid, Vitamin B12, fasting plasma glucose, fasting insulin, and HbA1c level.

**Table 1:** Demographic and baseline characteristics of participants.

	**Non–cognitive**	**Cognitive**	**P value**
	**frailty (*n* = 122)**	**frailty (*n* = 32)**	
**Demographics**			
**Age (years)** ^**a**^	68.0(65.0–72.0)	70.0(69.0–73.8)	**0.001**
**Gender**			0.212
Male (*n*, %)	53 (43.4)	10 (31.2)	
Female (*n*, %)	69 (56.6)	22 (68.8)	
**Duration of hypertension**			0.194
≤ 10 years (*n*, %)	80 (65.6)	17 (53.1)	
>10 years (*n*, %)	42 (34.4)	15 (46.9)	
**Duration of diabetes**			0.606
< 10 years (*n*, %)	71 (58.2)	17 (53.1)	
≥10 years (*n*, %)	51 (41.8)	15 (46.9)	
**Education level**			0.056
Primary or below (*n*, %)	73 (59.8)	25 (78.1)	
Secondary or above (*n*, %)	49 (40.2)	7 (21.9)	
**Income (RMB/month)**			**0.000**
< 1,000 (*n*, %)	60 (49.2)	27 (84.4)	
≥1,000 (*n*, %)	62 (50.8)	5 (15.6)	
**Occupation**			**0.012**
Retiree (*n*, %)	48 (39.3)	5 (15.6)	
Peasant (*n*, %)	74 (60.7)	27 (84.4)	
**Smoking**			0.532
Smoker (*n*, %)	21 (65.6)	11 (34.4)	
Non–smoker (*n*, %)	87 (71.3)	35 (28.7)	
**Drinking**			0.250
Yes (*n*, %)	23 (18.9)	9 (28.1)	
No (*n*, %)	99 (81.1)	23 (71.9)	
**Scales**			
MNA–SF^a^	11.0(11.0–13.0)	10.5(9.0–11.0)	**0.002**
GDS−15^a^	1.0(0.0–2.0)	2.0(0.0–4.0)	**0.037**
SSRS	39.6 ± 7.0	36.7 ± 5.9	**0.030**
ADL^a^	14.0(14.0–14.0)	15.0(15.0–18.0)	**0.000**
MFS^a^	35.0(35.0–35.0)	35.0(35.0–56.3)	**0.000**
**Laboratory indexes**			
Albumin (g/L)	45.4 ± 2.6	44.7 ± 3.3	0.296
Calcium (mmol/L)^a^	1.18(1.14–1.21)	1.17(1.13–1.22)	0.331
25–Vitamin D (ng/mL)^a^	20.1(15.5–24.5)	18.5(12.9–21.8)	0.120
Homocysteine (umol/L)^a^	17.9 ± 4.1	18.5 ± 4.0	0.507
Folic acid (nmol/L)^a^	31.4(23.7–41.9)	31.9(24.7–41.2)	0.883
Vitamin B12 (pmol/L)^a^	281.0(208.0–356.5)	285.0(174.3–400.8)	0.982
TG (mmol/L)^a^	1.46(1.11–2.36)	1.67(1.09–2.36)	0.956
LDL (mmol/L)^a^	2.16(1.75–2.86)	2.33(1.73–2.84)	0.640
HDL (mmol/L)^a^	1.05(0.85–1.34)	1.09(0.77–1.25)	0.745
FPG (mmol/L)^a^	7.9(6.8–9.1)	8.1(7.0–9.2)	0.513
FI (mU/L)^a^	10.8(7.3–16.7)	14.5(8.7–16.1)	0.181
HbA1C (%)^a^	6.7(6.0–7.9)	6.3(5.8–8.8)	0.167

MNA-SF, Mini-Nutrition Assessment-Short Form; GDS-15, Geriatric Depression Scale 15-item; SSRS, Social Support Rating Scale; ADL, Activities of Daily Living; MFS, Morse Fall Scale; TG: triglycerides, LDL, low-density lipoprotein cholesterol; HDL, high-density lipoprotein cholesterol; FPG, fasting plasma glucose; FI, fasting insulin. Data are presented as %(n), mean ±SD and median (25^th^-75th percentile). ^a^Compared by Mann-Whitney U test.

The bold values mean *p* < 0.05.

After adjusting for education level, status of nutrition, GDS-15, age and ADL score were risk factors for cognitive frailty. After adjusting for age, social support, ADL score, Morse Fall score, GDS-15 score, MNA-SF score, income, and occupation in Table 2, the risk of cognitive frailty increased significantly with age (OR = 1.164, 95% CI: 1.022–1.326, *p* < 0.05) and ADL score (OR = 1.308, 95% CI: 1.024–1.670, *p* < 0.05), and decreased significantly with monthly income (OR = 0.237, 95% CI: 0.059–0.955, *p* < 0.05).

**Table 2:** Multivariate regression analysis using cognitive frailty as the dependent variable.

**Model 1**	**Adjusted OR**	**P value**	**95% Confidence interval**	
			**Lower bound**	**Upper bound**
Age	1.151	**0.021**	1.021	1.298
Education level	0.422	0.110	0.147	1.215
MNA-SF	0.832	0.214	0.623	1.112
GDS-15	1.248	**0.049**	1.001	1.557
ADL	1.434	**0.004**	1.124	1.831
**Model 2**
Age	1.164	**0.022**	1.022	1.326
MNA-SF	0.867	0.360	0.640	1.176
Occupation	0.759	0.713	0.175	3.297
Income	0.237	**0.043**	0.059	0.955
MFS	1.037	0.218	0.979	1.100
SSRS	0.997	0.940	0.924	1.076
GDS-15	1.190	0.151	0.938	1.510
ADL	1.308	**0.032**	1.024	1.670

MNA-SF, Mini-Nutrition Assessment-Short Form; MFS, Morse Fall Scale; SSRS, Social Support Rating Scale; GDS-15, Geriatric Depression Scale 15-item; ADL, Activities of Daily Living. Adjusted OR, Adjusted odds ratio. Model 1: adjusted for age, education level, MNA-SF, GDS-15, ADL. Model 2: adjusted for age, MNA-SF, MFS, occupation, income, MFS, SSRS, GDS-15, ADL.

The bold values mean *p* < 0.05.

## Discussion

Recently, comprehensive geriatric assessment has been attracting increasing attention in China. In this study, we screened the associated factors of mild cognitive impairment and physical frailty in the elderly by reviewing relevant literature. We evaluated the nutritional status, depression level, social support, demographic and clinical characteristics, lipid levels, blood glucose levels, and other indicators of the recruited population. This was the first study to investigate the influencing factors of cognitive frailty in the elderly with hypertension and diabetes. Cognitive frailty can progress to various forms of dementia (25–27), thus increasing the risk of hospitalization, falls, and death (28–30). About 20.8% of the participants were determined to have cognitive frailty in this study, a number higher than that previously reported (31). This may be explained by the fact that the participants in this study had multiple chronic diseases. Mone et al. (32) suggest that hypertension and diabetes, which are associated with endothelial dysfunction, inflammation, and oxidative stress, can lead to cognitive frailty. Furthermore, adults with cognitive frailty in this study were older, had a lower income, and were mainly peasants with a high risk of malnutrition, less social support, higher ADL score, and a high risk of falling. Moreover, age, income, and ADL score were significantly associated with cognitive frailty, even after adjusting for other possible influencing factors.

Age was found to be the most common risk factor for cognitive frailty, which is consistent with the study of Kim et al. and Mone et al. (33, 34). High-income people may have a stronger awareness of chronic disease management and could intervene in the early stage of the disease. The terms physical frailty, cognitive impairment, and cognitive frailty have partially overlapped in definitions. Several studies have investigated the effects of ADL on people with cognitive frailty, but the assessment methods of ADL in other study are different. IADL has been shown as a risk factor for physical frailty and cognitive impairment. Research also shows that physical disability, indicated by lower performance in BADL (assessed by the Katz index), is a risk factor for cognitive frailty (11, 35–37). Ma et al. (38) showed that cognitive frailty can increase the risk of BADL disability by over ten times during a 3-year follow-up in the elderly compared to robust adults. As Avila-Funes et al. (39) supported, people with cognitive frailty tended to develop ADL dependence. They used internationally recognized scales to assess BADL and IADL dependence. In the present study, the risk of ADL dependence in participants with cognitive frailty was significantly greater than in those without cognitive frailty.

There was no statistical significance in the education level between the two groups in this study, which might be associated with the distribution of education level of our individuals. More participants were primary or lower education level in our study. However, after adjusted for education level, MNA-SF, GDS-15, age and ADL score still significantly increased risk of cognitive frailty. Previous studies suggested that cognitive frailty could be improved with nutritional interventions, muscle exercise, resistance training, and fall avoidance (17, 40). Our study also suggested that improving the ability to perform daily living activities may reduce the prevalence of cognitive frailty in the elderly population. Age is an irreversible factor. With increasing age, more attention should be paid to people with hypertension and diabetes at lower income levels and lower ability of daily living.

There are several limitations of this study. First, the study had a relatively small sample size. Future research should include a bigger sample population. Second, this was a cross-sectional study, and follow-up is required to confirm the findings. Finally, the groups of physical frailty and mild cognitive impairment only should be constructed.

Furthermore, we can screen people at a high risk of cognitive frailty based on ADL assessment quickly. Future research should focus on improving the level of ADL, which may be beneficial in reducing the prevalence of cognitive frailty.

## Conclusion

Cognitive frailty is correlated with age, monthly income, and ability to perform daily living activities in the elderly with hypertension and diabetes. For the elderly with low income and poor self-care ability, early intervention for cognitive frailty should be carried out. We will next investigate the effects of these interventions on early dementia.

## Data availability statement

The raw data supporting the conclusions of this article will be made available by the authors, without undue reservation.

## Ethics statement

The studies involving human participants were reviewed and approved by Ethics Committee of the Wuxi People's Hospital. The patients/participants provided their written informed consent to participate in this study.

## Author contributions

KH, XZ, YZ, and SL designed the research. SL, QX, JY, YY, ZW, BZ, SW, and XC helped with the data collection and analysis. SL and QX wrote the first draft of the manuscript. KH, XZ, and YZ contributed to the critical revision of the manuscript. All authors contributed to the manuscript revision and approved the submitted version.

## Funding

This work was funded by the Young Project of the Wuxi Health Committee (Q201914), the Top Talent Support Program for Young and Middle-Aged People of Wuxi Health (BJ2020008), and the Major Project of the Wuxi Health Committee (Z202002).

## Conflict of interest

The authors declare that the research was conducted in the absence of any commercial or financial relationships that could be construed as a potential conflict of interest. The reviewer XE declared a shared affiliation with the authors SL, QX, JY, YY, ZW, BZ, YZ, XZ, and KH to the handling editor at the time of review.

## Publisher's note

All claims expressed in this article are solely those of the authors and do not necessarily represent those of their affiliated organizations, or those of the publisher, the editors and the reviewers. Any product that may be evaluated in this article, or claim that may be made by its manufacturer, is not guaranteed or endorsed by the publisher.
